# The bidirectional relationship between children’s bedtime irregularity & hyperactivity/inattention symptoms: a prospective cohort study

**DOI:** 10.1186/s40359-026-04192-3

**Published:** 2026-03-09

**Authors:** Kin Ho Alan Li, Mark Lawrence Wong

**Affiliations:** Department of Social and Behavioural Sciences, City University of Hong Kong, Tat Chee Avenue, Kowloon Tong, Hong Kong Hong Kong SAR

**Keywords:** Bedtime irregularity, Hyperactivity/ inattention, Neurodiversity, Good health and well-being, Child development

## Abstract

**Objectives:**

Although cross-sectional data indicated positive association between hyperactivity and inattention problems and irregular bedtime, longitudinal studies on this topic are scarce leading to no clear inference regarding their directional relationship. Thus, using a prospective birth cohort, we examined the longitudinal relationship between hyperactivity/inattention problems and bedtime irregularity among children aged 5–11 years.

**Study design:**

We made use of a nationally representative British birth cohort (*N* = 15430). Parents reported their children’s hyperactivity/inattention symptoms and bedtime irregularity when the children were aged 5, 7 and 11 years. Data were analyzed using the cross-lagged autoregressive model.

**Results:**

Hyperactivity/inattention symptoms (*β*s = 0.74, *p*s < 0.001) and bedtime irregularity (0.35 ≤ *β*s ≤ 0.45, *p*s < 0.001) remained stable across the 6-year period. Hyperactivity/inattention symptoms and bedtime irregularity consistently predicted each other over time (0.02 ≤ *β*s ≤ 0.05, *ps < .*05).

**Conclusions:**

Our findings supported a small, yet statistically significant bidirectional relationship between hyperactivity/inattention symptoms and bedtime irregularity during childhood. Findings are consistent with the possibility that regularising bedtime may be associated with improvements on children’s hyperactivity/inattention behaviour. Targeting both hyperactivity/inattention symptoms and bedtime irregularity might be a better approach to optimise overall functioning.

**Supplementary Information:**

The online version contains supplementary material available at 10.1186/s40359-026-04192-3.

## Introduction

Sleep has a vital role in optimising healthy development among school-aged children, and attending formal schooling is one of the first major environmental factors affecting children’s sleep-wake schedule. Over 25% of school-aged children struggled to maintain a stable bedtime routine [1, 2]. Results from regionally representative data showed that regularity of bedtime was correlated with hyperactivity/inattention (H/I) symptoms among neurotypical children [3, 4]. Yet, to date, the directional relationship between bedtime regulation and H/I symptoms is unclear with the lack of longitudinal or experimental data.

Sleep problem is widely recognized among individuals with Attention-Deficit/Hyperactivity Disorder (ADHD) [5]. However, sleep problem embodies a multitude of concepts which bedtime regularity may be an important but less understood sleep-related parameter. Bedtime irregularity refers to having inconsistent timing to go to bed across days in a week. Cross-sectional studies found that individuals with ADHD had increased chance of having irregular sleep-wake schedule, relative to those without ADHD [6, 7]. Among neurotypical children sample, bedtime irregularity, was also found to predict poorer performance on neurobehavioral tasks requiring attention and inhibition [8]. A community-based study found that bedtime irregularity was strongly associated with parent-reported H/I symptoms in neurotypical children [3]. While the abovementioned data is correlational, there are mixed opinions regarding the directional relationship between H/I symptoms and bedtime irregularity. Some researchers suggest that bedtime irregularity contributes to difficulties in sustaining attention and inhibiting impulsivity [9, 10], where bedtime irregularity may preclude children from having healthy sleep and affect their brain development, which result in attention deficits and hyperactivity. Comparatively, others suggest [3, 6, 11] bedtime irregularity is a nighttime manifestation of daytime behavioral difficulties. A third perspective is that there is a bidirectional relationship between bedtime irregularity and H/I symptoms [12]. This interpretation aligns with the *developmental cascade model* [13] which specifies development as the cumulative consequences of interactions and transactions within and across different domains, rather than being construed as unfolding in a linear fashion across development [13]. Based on this perspective, problematic behaviours that occur at the earlier course of development may have a subtle link with the within- and/ or across-domains of behaviours that occur further downstream. Previous studies have established the autoregressive effects of H/I symptoms [14] and bedtime irregularity among children [15]. However, they did not depict the interrelations between across-domain behaviours across developmental stages. Therefore, longitudinal data which examine the directional relationship between bedtime irregularity and H/I symptoms across time is needed to further understand the complexity of the association.

### The current study

Using a nationally representative prospective British birth cohort, we aim to model the change trajectory of bedtime irregularity and H/I symptoms as well as their inter-relationships at 3 time points (age 5, 7 and 11). Given the increasing recognition that inattention and hyperactivity problem is on a spectrum [16, 17], we adopt a dimensional approach to assess H/I symptoms and bedtime irregularity [18]. Based on existing cross-sectional findings, we hypothesized a bidirectional relationship between bedtime regularity with H/I symptoms.

## Methodology

### Study design

This study used a secondary quantitative research approach, using data drawn from the Millennium Cohort Study (MCS) [19], a prospective population-based cohort study of more than 18,000 families that have at least one child born between 2000 and 2002 across the nations in the United Kingdom [19]. Data from Wave 3 through Wave 5 were used when children were around 5, 7 and 11 years old. The report of this prospective cohort study was written in compliance with the STROBE (Strengthening the reporting of observational studies in epidemiology) statement – checklist for cohort studies.

### Participants

**Fig. 1:**
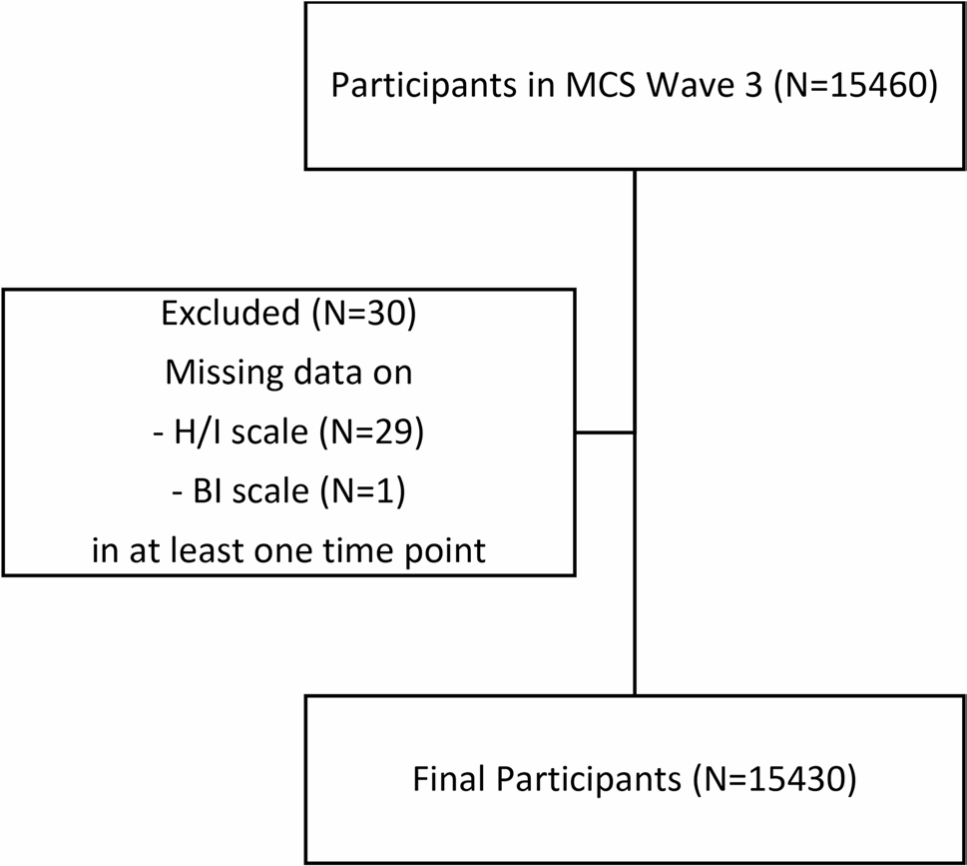
Flowchart illustrating exclusion details from the final analysis. Notes: H/I – Hyperactivity/inattention symptoms; BI – Bedtime irregularity

The inclusion criteria included (1) valid response on the H/I symptoms measure for at least one measurement point, and (2) valid response on the rating of bedtime irregularity for at least one measurement point (Fig. 1). In MCS, 18,818 participants started the study when they were at 9 months old [19]. 15,460 children continued to participate when they were age 5, where 15,430 children (82.0% of original sample) fulfilled the inclusion criteria and were included for data analysis in this study (Table 1**)**. The number of individuals included in the analyses at each time point: age 5 (13815), age 7 (12901) and age 11 (11694).

**Table 1:** Descriptive statistics

	Mean / Percentage (SD)
Gender (Female : Male)	7553 : 7877
Ethnicity	
- White	12,866 (83.4%)
- Mixed	443 (2.9%)
- Indian	385 (2.5%)
- Pakistani	697 (4.5%)
- Bangladeshi	293 (1.9%)
- Chinese	22 (0.1%)
- Black Caribbean	176 (1.1%)
- Black African	312 (2.0%)
- Other Ethnic Group	232 (1.5%)
Household weekly income (GBP)	345.50 (217.10)
Parent’s highest educational level	
- NVQ level 1	1818 (7.1%)
- NVQ level 2	7062 (27.5%)
- NVQ level 3	3772 (14.7%)
- NVQ level 4	7501 (29.2%)
- NVQ level 5	1305 (5.1%)
- Others	4261 (16.6%)
5y H/I symptoms	8.26 (2.37)
7y H/I symptoms	8.33 (2.51)
11y H/I symptoms	8.11 (2.48)
5y Bedtime irregularity	1.53 (0.81)
7y Bedtime irregularity	1.53 (0.77)
11y Bedtime irregularity	1.57 (0.77)

Notes: *H/I* Hyperactivity/inattention symptoms, *BI* Bedtime irregularity, *GBP* British Pound Sterling, *NVQ* National vocational qualification

### Measures

H/I symptoms were assessed by H/I subscale in the Strengths and Difficulties Questionnaire (SDQ) [20]. Parents responded to the items based on the children’s behavior over the last six months. The H/I subscale contained five items (e.g., “restless”) and each was rated on a 3-point scale, with higher scores indicating higher H/I symptoms. The sum of the 5 items formed the H/I subscale score, which was used in this study to represent children’s H/I symptoms. The H/I subscale had good internal consistency among the study’s sample across the 3 time-points (Cronbach α_5y_ = 0.77, α_7y_ = 0.79, α_11y_ = 0.79).

Bedtime Irregularity was assessed by a single parent-reported item which asked, “On weekdays during term-time, does your child go to bed at regular time?”. The parent responded on a 4-point scale (1 = never, 2 = sometimes, 3 = usually, and 4 = always). In the current study, the values of the response were reversed-coded to represent the extent of sleep irregularity of the cohort members. As such, higher scores indicate a more irregular bedtime.

### Data analytic approach

The concurrent association between bedtime regularity and H/I symptoms was assessed by Spearman’s correlation, given that all variables were ordinal values and the data did not follow normal distribution (Supplementary Table 1). The longitudinal associations among the variables were assessed by an autoregressive cross-lagged model with the Diagonally Weighted Least Square (DWLS) estimation for non-normal and ordinal data [21]. Missing data were handled using pairwise deletion, where each correlation or covariance was calculated using all available data for that specific pair of variables. To assess the first-order autoregressive pathways, each study variable was specified as a predictor of its own value at the subsequent time point. Therefore, autoregressive pathways estimate the unidirectional association between the variable at n_year_ and the same variable at later age. In line with previous research, the autoregressive pathways were allowed to vary across time [14]. For the cross-lagged pathways, a variable was presented as either a predictor or an outcome of another variable over time. Therefore, cross-lagged pathways estimate bidirectional associations between one variable at n_year_ and another variable at later age. Likewise, these effects were allowed to vary across time [14]. Model fit of the hypothesized model was evaluated with the following fit indices criteria, e.g., Tucker–Lewis Index (TLI) and the Comparative Fit Index (CFI) > 0.95; Root Mean Square Error of Approximation (RMSEA) < 0.05 [22]. Because the Chi-Square value (χ^2^) becomes increasingly sensitive as sample size grows [23], it is presented for completeness but not used to evaluate model fit. Standardized regression coefficients (β) were to examine the effect size of the relationship. All data analyses were conducted via correlation analysis and path analysis in a structural equation modelling (SEM) framework, using the statistical software Jamovi [24].

## Result

The concurrent correlation analyses revealed positive and small correlations between higher levels of H/I symptoms and higher levels of bedtime irregularity from age 5 through 11, *r*_5y_=0.088, *r*_7y_=0.066, *r*_11y_=0.065, *p*s < 0.001.

### Autoregressive pathways

**Fig. 2:**
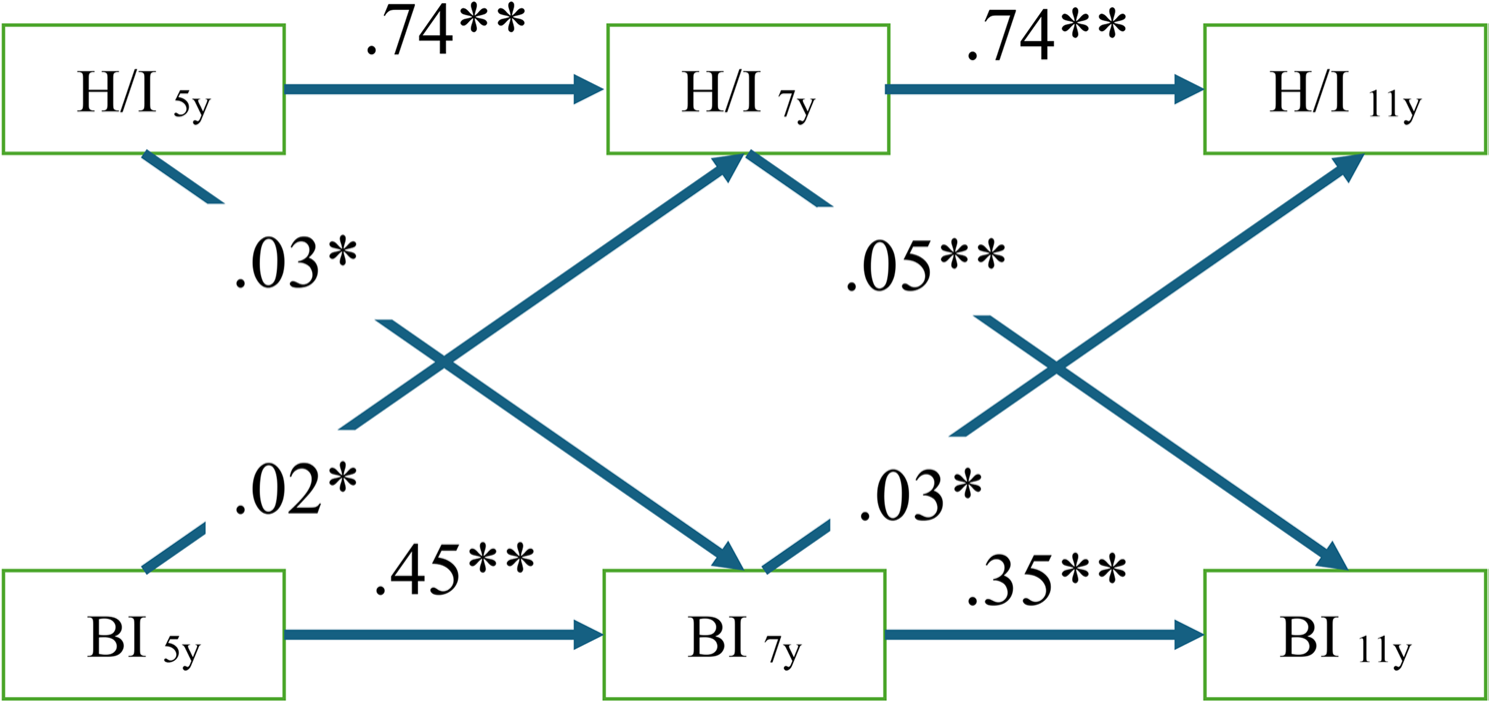
Significant autoregressive and cross-lagged paths from age 5 through 11 are depicted. Overall Model Fits: TLI = .98; CFI = .99; RMSEA =.04(.03-.05). H/I – Hyperactivity/inattention symptoms; BI – Bedtime irregularity; TLI – Tucker–Lewis Index; CFI – Comparative Fit Index; RMSEA – Root Mean Square Error of Approximation. * *p* <.05; ** *p* < .01; *** *p* <.001

The hypothesized model was found to have a good fit with the data (TLI = 0.98, CFI = 0.99, RMSEA = 0.04, 95% CI(0.03-0.05)) (Fig. 2). The autoregressive associations were significant from age 5 through 11 with respect to H/I symptoms with strong effect sizes (*β*s = 0.74, *p*s < 0.001) and bedtime irregularity with medium effect sizes (0.35 ≤ *β*s ≤ 0.45, *p*s < 0.001), where higher levels of H/I symptoms and bedtime irregularity at earlier age consistently predicted higher levels of the same at later age (Fig. 2; Table 2).

**Table 2:** Path analyses between Hyperactivity/Inattention symptoms with bedtime irregularity across time

Paths	β	*p*
H/I_5y_ → H/I_7y_	0.74	< 0.001***
H/I_7y_ → H/I_11y_	0.74	< 0.001***
H/I_5y_ → Bedtime Irregularity_7y_	0.03	0.002**
H/I_7y_ → Bedtime Irregularity _11y_	0.05	< 0.001***
Bedtime Irregularity _5y_ → Bedtime Irregularity _7y_	0.45	< 0.001***
Bedtime Irregularity _7y_ → Bedtime Irregularity _11y_	0.35	< 0.001***
Bedtime Irregularity _5y_ → H/I_7y_	0.02	0.014*
Bedtime Irregularity _7y_ → H/I_11y_	0.03	0.018*

Notes: *β* standardized auto-regressive paths, *H/I* Hyperactivity/inattention symptoms, *BI* Bedtime irregularity. *p < .05; **p < .01; ***p < .001

### Cross-lagged pathways

Higher levels of H/I symptoms were consistently linked to higher levels of bedtime irregularity from age 5 through 11 (0.03 ≤ *β*s ≤ 0.05, *ps* ≤ 0.01). Similarly, Higher levels of bedtime irregularity were consistently linked to higher levels of H/I symptoms from age 5 through 11 (0.02 ≤ *β*s ≤ 0.03, *ps < .*05). (Fig. 2; Table 2). In response to the reviewer’s suggestion, we tested an exploratory alternative model which included 4 additional pathways which depicted the effects of age 5 H/I symptoms and bedtime irregularity on age 11 H/I symptoms and irregularity (Supplementary Table 1). We here preferred the original model because of parsimony and also the potential collinearity issues between age 5 and age 7 measures in predicting age 11 measures.

## Discussion

The current study investigated the longitudinal relationship between bedtime irregularity and H/I symptoms among children from age 5 through age 11 with a nationally representative prospective British cohort. Consistent with the hypotheses, our findings showed significant concurrent associations between H/I symptoms and bedtime irregularity. In addition, both bedtime irregularity and H/I symptoms were stable over time. This result added to existing evidence that H/I symptoms and bedtime irregularity follow a developmental process where early behaviour could link to later development [14, 15]. Our findings additionally revealed a stable, yet small bidirectional relationship between bedtime irregularity and H/I symptoms in school-aged children. The significant bidirectional relationship might be explained by the Developmental Cascade Model [13], where H/I symptoms and bedtime irregularity underwent a complex developmental process through autoregressive and cascading effects across the developmental course. An alternative interpretation may be that their association were related to common biologically rooted mechanism – the Prefrontal Cortex (PFC). The PFC subserved executive functions such as impulse control and sustained attention, which were hallmark features of H/I symptoms, and sleep/wake regulation [25]. Therefore, severe H/I symptoms and bedtime irregularity might reflect suboptimal PFC functioning and behavioural disturbances [26]. However, the effects observed were relatively small, suggesting that while the relationship is plausible, its practical implications should be interpreted with caution. Given that bedtime irregularity was only assessed by a single item, future studies with more comprehensive measures of bedtime regularity, e.g. sleep diary, actigraphy, would be helpful to further ascertain the prospective association. Still, the current findings offered a solid empirical basis for further clinical or intervention study on bedtime regularity and H/I symptoms. A better understanding of the pattern and mechanism of change between bedtime regularity and H/I symptoms during childhood may inform timely and early identification of children at risk and foster development for appropriate intervention.

### Limitations

A major limitation of this study was the lack of a multi-informant perspective approach, as parents were the only respondents. The two core measures (bedtime irregularity and H/I symptoms) were both rated by parents only, contributed to artificial inflation or deflation of correlations between the variables. Also, the large sample size in this study increased statistical power, which in turn raised the likelihood of detecting associations that be trivial in real-world terms. Therefore, further investigation of the prospective association between H/I symptoms and bedtime regularity using different measures and design, including teacher reports and objective measures of sleep, were needed [14]. In addition, the small effect sizes found might indicate that there were possibly unexplored factors (e.g., household functioning) [3] which contributed to the development of these behaviours. Also, currently we handled missing data by pairwise deletion, where the results were generated based on individuals with valid data on the variables concerned. Future studies with different prospective dataset using different missing data analysis technique could help to further ascertain the prospective association among bedtime regularity and H/I symptoms. Despite the limitations, the current study had its major strengths with the use of a sizable nationally representative birth cohort which allowed assessment of the complex associations between H/I symptoms and bedtime regularity across 3 time points.

## Conclusions

In conclusion, our results indicated that bedtime irregularity and H/I symptoms tended to occur concurrently and they persisted from age 5 to age 11. Additionally, this study found novel evidence of the stable bidirectional relationship between bedtime irregularity and hyperactivity/inattention symptoms. The findings altogether provided solid evidence supporting the possibility that regularising bedtime might be associated with changes on H/I symptoms. It also highlighted the needs to target both bedtime irregularity and H/I symptoms to optimise healthy childhood development.

## Supplementary Information

Supplementary Material 1.

Supplementary Material 2.

## Data Availability

There is no new data or materials generated from this secondary data analysis study.

## Abbreviations

ADHD: Attention-Deficit/Hyperactivity Disorder
α_5y_ / α_7y_ / α_11y_: Cronbach’s α at age 5/7/11
b: Standardized regression coefficient
CFI: Comparative Fit Index
CI: Confidence Interval
DWLS: Diagonally Weighted Least Squares
H/I: Hyperactivity/Inattention
MCS: Millennium Cohort Study
PFC: Prefrontal Cortex
r_5y_/ r_7y/_ r_11y_: Correlation Coefficients at age 5/7/11
RMSEA: Root Mean Square Error of Approximation
SDQ: Strengths and Difficulties Questionnaire
SEM: Structural Equation Modelling
TLI: Tucker–Lewis Index
