# Supplementary material for: The bidirectional relationship between children’s bedtime irregularity & hyperactivity/inattention symptoms: a prospective cohort study
Source: BMC Psychol. 2026 Mar 9;14:536. doi: 10.1186/s40359-026-04192-3 (PMC13081608; doi:10.1186/s40359-026-04192-3)
Supplement: Supplementary file 2 — Supplementary Material 2. [file 40359_2026_4192_MOESM2_ESM.docx]

| *Supplementary Table 1.* Path Analyses between Hyperactivity/Inattention Symptoms with Bedtime Irregularity across time | | |
| --- | --- | --- |
| Paths | β | *p* |
| H/I_5y_ → H/I_7y_ | .70 | <.001*** |
| H/I_7y_ → H/I_11y_ | .53 | <.001*** |
| H/I_5y_ → H/I_11y_ | .22 | <.001*** |
| H/I_5y_ → Bedtime Irregularity_7y_ | .04 | <.001*** |
| H/I_7y_ → Bedtime Irregularity _11y_ | .02 | .434 |
| H/I_5y_ → Bedtime Irregularity_11y_ | .02 | .394 |
| Bedtime Irregularity _5y_ → Bedtime Irregularity _7y_ | .40 | <.001*** |
| Bedtime Irregularity _7y_ → Bedtime Irregularity _11y_ | .24 | <.001*** |
| Bedtime Irregularity _5y_ → Bedtime Irregularity _11y_ | .15 | <.001*** |
| Bedtime Irregularity _5y_ → H/I_7y_ | .02 | .043* |
| Bedtime Irregularity _7y_ → H/I_11y_ | .02 | .283 |
| Bedtime Irregularity _5y_ → H/I_11y_ | .02 | .395 |
| Notes: β – standardized auto-regressive paths; H/I – Hyperactivity/inattention symptoms; BI – Bedtime irregularity. **p* <.05; ***p* < .01; ****p* <.001 | | |
